# A Comparative Study of Registration Methods for RGB-D Video of Static Scenes

**DOI:** 10.3390/s140508547

**Published:** 2014-05-15

**Authors:** Vicente Morell-Gimenez, Marcelo Saval-Calvo, Jorge Azorin-Lopez, Jose Garcia-Rodriguez, Miguel Cazorla, Sergio Orts-Escolano, Andres Fuster-Guillo

**Affiliations:** Institute for Computing Research, University of Alicante, P.O. Box 99, Alicante, Spain; E-Mails: vmorell@dccia.ua.es (V.M.-G.); msaval@dtic.ua.es (M.S.-C.); jazorin@dtic.ua.es (J.A.-L.); jgarcia@dtic.ua.es (J.G.-R.); sorts@dtic.ua.es (S.O.-E.); fuster@dtic.ua.es (A.F.-G.)

**Keywords:** RGB-D sensor, registration, robotics mapping, object reconstruction, **PACS** 42.30.Tz

## Abstract

The use of RGB-D sensors for mapping and recognition tasks in robotics or, in general, for virtual reconstruction has increased in recent years. The key aspect of these kinds of sensors is that they provide both depth and color information using the same device. In this paper, we present a comparative analysis of the most important methods used in the literature for the registration of subsequent RGB-D video frames in static scenarios. The analysis begins by explaining the characteristics of the registration problem, dividing it into two representative applications: scene modeling and object reconstruction. Then, a detailed experimentation is carried out to determine the behavior of the different methods depending on the application. For both applications, we used standard datasets and a new one built for object reconstruction.

## 1. Introduction

Registration of multiple 3D datasets is a fundamental problem in many areas, such as computer vision, medical imaging [1], object reconstruction, mobile robotics, augmented reality [2], *etc*. Due to the increasing usage of current low-cost RGB-D sensors, this technology has opened new lines of research.

Three-dimensional data can be obtained from different devices: 3D lasers, stereo cameras, time-of-flight cameras, RGB-D cameras, *etc*. Depending on the input sensor, some algorithms provide better results than others. 3D lasers are usually active, non-contact sensors. They emit a pulse of light and measure the time spent by the light to return to the device. Others 3D lasers use a triangulation using a camera to measure the deviation of the light depending on the depth of the object from which the light was reflected. Some 3D laser systems do not provide color information, so algorithms that need visual features are not suitable. Other 3D lasers systems provide color information (using different approaches to incorporate color to the depth information), but their cost is prohibitive. Stereo cameras use two or more conventional cameras to obtain the disparity (the difference of position from one camera to the others, usually by correlation) and, from it, the depth (objects close to the camera have less disparity than farther ones). However, stereo cameras suffer from the lack of textures: image areas without texture do not provide depth information. Another sensor is the Photonic Mixer Device (PMD) (also known as time-of-flight cameras), which measures distances directly for a two-dimensional field of pixels, based on the time of flight of modulated infrared light. The visual information of PMD cameras, like SR4000, is infrared. It is affected by natural light and, normally, is noisy. In our previous work [3], we performed some experiments using the SIFT visual feature method [4] with this kind of camera. As the SR4000 camera provided noisy images, the repeatability of the SIFT feature was low.

Low-cost RGB-D sensors, such as Microsoft Kinect, Primesense Carmine (http://www.primesense.com) or Asus Xtion (http://www.asus.com/Multimedia/Xtion PRO), introduce a great advance to the robotics area. They are composed of two sensors: an IR (infrared) projector, an IR CMOS camera and an RGB camera. The IR sensor provides the depth information. The IR projector sends out a fixed pattern of bright and dark speckles. Using structured light techniques; depth is calculated by triangulation against a known pattern from the projector. The pattern is memorized at a known depth, and then, for each pixel, a correlation between a known pattern and the current pattern is done, providing the current depth at this pixel. In this work, we have used the Kinect camera for experiments. The Kinect camera has a resolution of 640 × 480 (307,200 pixels) and a working range between 1 and 8 m, approximately providing a frame rate up to 30 fps. A detailed analysis of the accuracy and resolution of this camera can be found in [5].

In this paper, we are focusing on low-cost RGB-D sensors. For the rest of the paper, when we mention RGB-D sensors or data, we are referring to the low-cost ones. With these specific kinds of sensors, we are interested in the study of the behavior of different registration methods for incremental video reconstruction of scenes and small objects. The work focuses on the registration of subsequent frames of a slowly moving Kinect camera in a static Lambertian scene.

The registration problem could be addressed in two ways. First is searching the solution in the correspondence space. In this case, the problem is comprised of two related sub-problems: correspondence selection and motion (or transformation) estimation. In the former, candidate correspondences between datasets are chosen, while in the latter, transformation minimizing the distances between corresponding points are estimated. Second is searching the solution in the transformation space. An objective function is defined (for example, the distance between two datasets), and a search using different transformations to find the transformation that minimize the objective function is performed.

Several reviews related to the registration problem can be found in the literature. In [6], a complete color image registration survey is presented. Tam *et al.* [7] made a survey of registration methods for rigid and non-rigid point clouds and meshes. In [8], a comparison among different iterative closest point (ICP) methods is presented, while in [9], a similar study is proposed, but with real-world datasets.

In this paper, we are focused on an experimental review of state-of-the-art rigid registration methods using RGB-D images. Therefore, our main contributions are:

- A study of the most used approaches to register static environments using RGB-D sensors and testing different methods using a state-of-the-art dataset.
- A study of the current methods for object reconstruction and a discussion of the problems of registering small objects using low-cost RGB-D sensors and also the creation of a new real-world dataset.

With respect to registration methods, RANSAC (random sample consensus) [10] usually works with features (visual or 3D). Since the global properties of objects are vulnerable to occlusions and clutter in the scene [11], local invariant features are used for this purpose. Moreover, local features could be used with non-rigid objects in scenarios, *i.e.*, articulated or deformable objects. RANSAC is faster than other methods, and it allows a proper registration in the presence of noisy data. However, it depends on the ratio between inliers and outliers. If there are many more outliers than inliers and the number of inliers is low, the probability of finding the best solution is low. Furthermore, if the number of matched features is low, there is a high probability of obtaining a small number of inliers. Iterative closest point (ICP [12,13]) uses all of the points in the scene. ICP needs an initial alignment to register the scene. However, for small and smooth camera or scene motion in rigid scenes, incremental methods, such as ICP, achieve good results. ICP is more suitable for the local motion of noisy surfaces, while RANSAC achieves better results for global motion with precise correspondences containing outliers.

There exist several variants of the ICP method that are more quickly calculated by reducing the amount of points or by extracting less features. In this work, we have compared the original ICP only considering a kd-tree to speed-up correspondence searching. Due to the high variety of methods, we have made a study of the registration methods that use RGB-D sensors in static scenarios.

In order to review and describe the state-of-the-art of the rigid registration approaches, we decided to classify them into coarse and fine methods.

Following the definition of Salvi *et al.* [14], coarse and fine registration mainly differs in the accuracy of the provided solution. Coarse registration aims at computing an initial estimation of the rigid motion between data points. The robustness of these methods may highly vary in measure, where in theory, low accuracy increases their speed. Most of coarse registration methods are iterative, despite the existence of linear approaches. It is important to highlight that many coarse approaches use a subset of the data (downsampling or keypoints) in order to reduce the computational cost.

Fine registration, on the other hand, is focused on providing the most accurate solution. These methods generally use a roughly initial estimation to unify all views in a common coordinate system (avoiding falling in local minima) and then refining the initial solution.

In [14], a table is presented where important aspects of coarse and fine registration methods are classified: kind of correspondences, motion estimation, robustness and registration strategy.

This classification is used in [14,15], but many others can be found in the literature, such as dense/sparse, intrinsic/extrinsic, *etc*. Despite this classification, most of the registration methods use a hybrid approach to firstly coarse register pre-aligning the data into a global coordinate system and, next, refining the result using fine registration methods. In order to analyze the performance and accuracy of the reviewed methods, we have divided the experiments into two categories. The first one is the scene reconstruction. It is often used to perform map building. The mapping problem consists of registering the point sets obtained by the robot at different positions in order to get a map of the environment around the robot. The second one is the object reconstruction, very similar to the previous one, but focused on object reconstruction, like sculptures, tools, plants, *etc*. We will develop a detailed experimentation to determine the best methods to solve both problems. This paper does not intend to be an exhaustive review of the state-of-the-art, but a comparative study among different approaches to estimate registration, tested in two important and representative applications in which registration is used as a part of the general process: scene mapping and object reconstruction. These applications cover most of the expected requirements for the output of a registration process. Specifically, they allow one to reach important conclusions about the performance of registration methods using RGB-D sensors.

The remainder of this paper is organized as follows; Section 2 presents the review of the state-of-the-art, presenting first the registration problem. Then, the two frameworks used to make the comparison (scene and object reconstruction) and the metrics used to compare them are presented in Section 3. Results are presented, and a discussion is given in Section 4. Finally, conclusions are drawn in Section 5.

## 2. Related Work

**Figure 1.**
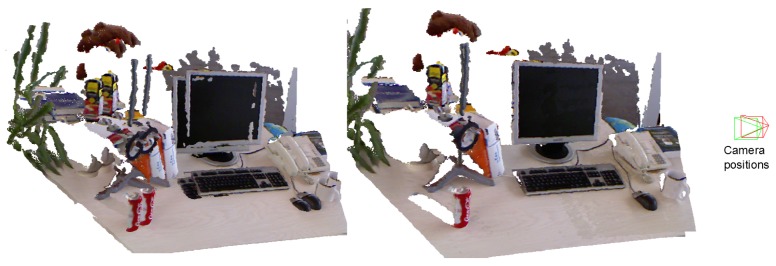
**(Left)** Two non-aligned point clouds. (**Right**) Both point clouds aligned and the estimated and reference positions of the camera.

Since registration has played an important role in many areas (including computer vision, medical imaging, image processing, etc), this task assumes different requirements depending on the scope. It could be analyzed from different perspectives. In medical imaging, it is usual to register images acquired by different kind of sensors, for example mapping an MRI image onto a CT scan. In image processing, registration is used to match images taken, for example, at different times or from different viewpoints. In general, it could be analyzed as the process of aligning different sets of data into one coordinate system. Figure 1 shows an example of a scene registration. On the left, we show the two point clouds provided by the Kinect camera, which are shown in a common reference system. As both point clouds were taken from different view points, objects appear repeated. On the right, we show both registered point clouds together with the initial position (red pyramid) and the estimated one (green pyramid) of the camera.

Irrespectively, if the registration task is considered as a mapping, matching or aligning process of sets of data, data are usually a set of two-dimensional (e.g., images) or three-dimensional points. In this paper, we are interested in the registration process aimed at aligning a set of 3D points. Therefore, we can define the registration problem approached in this work as the process of transforming different sets of 3D data into the same coordinate system. This means finding the transformation needed to align one new dataset, *S*, to a reference dataset, *M*.

Formally, the transformation, *T*, that minimizes the distance between the transformed points *S* = {*s*} and the reference points *M* = {*m*} is obtained by minimizing:

(1) $$T*=\arg\underset{T\in V}{\min}\sum_{s\in S}\sum_{m\in M}w_{sm}||m-T(s)||$$

where *m* is the point that matches with the point, *s*, ‖ · ‖ is the distance measure between points, usually euclidean distance, *V* = {*T*} is the set of all the possible transformations and *w_sm_* is the probability that the point, *s*, matches with the point, *m*. The problem can be simplified when the correspondence pairs between scene and model are known,

(2) $$T*=\arg\underset{T\in V}{\min}\sum_{i=1}^{N}\left||m_{i}-T(s_{i})|\right|$$

where *N* is the number of correspondence pairs and *m_i_* is the point of the model set which has a correspondence with the scene point, *s_i_*.

According to the transformation, *T*, applied to the set, *S*, rigid and non-rigid registrations could be obtained. A rigid registration transform the *S* points by a rotation and a translation preserving the distance between every pair of points in *S*. Rigid transformations are usually applied to static scenarios. However, non-rigid registrations are obtained to align the set, *S*, onto the reference points, *M*, when both datasets have non-linear geometric differences. In this case, non-rigid registrations are used to register deformations of objects (e.g., elastic surface deformation of an object) or articulated objects (e.g., human body movements). An interesting review could be found in the work written by Crum *et al.* [16]. As we are focused on static scenarios, non-rigid transformations are out of the scope of this paper.

According to the set of points to be registered, a wide or small baseline registration problem has to be dealt with. For wide baseline settings, the set of points, *M* and *S*, have been acquired from very different viewpoints (position and orientation of the camera) having a few overlap data between them. Registering laser scans from entirely different positions or registering two separate object reconstructions with a hand-held Kinect are examples of wide-baseline requirements for registration. Usually, methods try to detect invariant features from data to match the datasets using descriptors that look the same when a transformation, *T*, is applied. Additionally, other robust features are calculated (e.g., robust with respect to the lighting) that use only a local image region, so that feature-based registrations can work also with large parts of the dataset occluded, their relative order swapped, with dynamic objects, *etc.* On the other hand, small baseline settings assume data from close viewpoints having a larger overlapped areas in both datasets. This data can be obtained from a 3D image or a video acquisition system. For video acquisition systems, registration is usually carried out frame to frame. Depending on the frame rate, sometimes registration can deal with neglectable video motion, having very close viewpoints between frames. As a consequence, all those above feature properties for wide-baseline registration are useless in subsequent video frames of static scenes, because everything looks almost identical.

Another important aspect related to the set of points to be registered is the source of the points that conform the datasets, *S* and *M*. In general, *S* and *M* contains points from a viewpoint. Registration of two single views is usually considered a pairwise registration. When more than two views are taken into account into the process, a multiview registration is performed. In this case, the dataset, *M*, contains more than one view. It could contain a meta-view composed of raw views or a model of the scene previously registered being able to perform an incremental registration, aligning new views to the model, even already registered views can be registered again.

Applications discussed in this comparative study (mapping for robotics and small object reconstruction) of registration methods using RGB-D video deal with static scenarios. Hence, we are going to focus on rigid registration methods. Moreover, since the low-cost RGB-D cameras allow the capture of reasonably accurate mid-resolution depth and appearance information at high frame rates [17], the set of points to be registered have small baseline settings. As a consequence, the problem of rigid registration for small baseline settings using RGB-D video is going to be analyzed.

**Figure 2.**
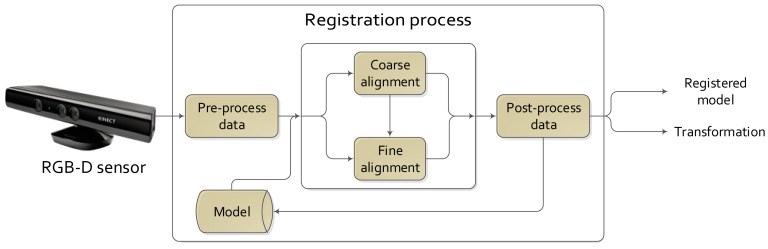
General scheme of the registration process.

There are several approaches to solve the frame-to-frame registration problem in RGBD video sequences, but all of them could be fitted in a general scheme. Figure 2 shows the main steps of a complete registration process. These steps are:

- Pre- and post-processing are phases where smoothing or noise rejection techniques are applied to enhance the result of the registration.
- The model data source is the module where the information from the output of the process is stored. It could contain the previous data and/or the obtained transformations. These data could be used to obtain the current registration: the previous data (3D data) as the current model; the previous estimated transformation as the initial transformation as, e.g., for the ICP.
- The output of the process could be just the transformation or the registered model.

However, not always are these steps carried out. Focusing on the coarse and fine registration phases, the preprocessing phase could be omitted when views are close enough to each other, and an initial alignment step is not needed. The post-processing phase could be avoided if the demanded accuracy is not high and accurate enough results were provided by the coarse registration phase. Furthermore, there exists different techniques to apply the registration process, such as multi-view, where one dataset is registered against a group of datasets and not only against a single view.

Regarding time performance, coarse registration methods could be faster than fine methods, due to them not needing to find the best solution, as well as the use of features from the scene, reducing the amount of data to be registered. However, they need additional time to calculate features and to match them. Generally speaking, generic algorithms that were conceived of to be used with different 3D sensors (stereo, laser, *etc*), e.g., ICP for fine registration and RANSAC for coarse registration, have not been developed considering temporal constraints. Despite the ICP having several variants, including the use of a kd-tree to search the closest pair of points or reducing the number of noises by rejecting wrong data, even uniformly downsampling the original data, it is still a slow algorithm. Furthermore, RANSAC is time consuming in those cases where the number of tested random samples is too large, and then, several iterations of the algorithm have to be executed. However, implementations able to work at video frequency can be found in the literature for both methods [8,18]. Specific methods designed for RGBD cameras, including ICP for refinement, as KinectFusion [19], dense visual odometry (DVO) [20] and RGBDemo [21], were developed to work with cameras that provide about 30 fps, such as the Microsoft Kinect.

### 2.1. Coarse Methods

**Figure 3.**
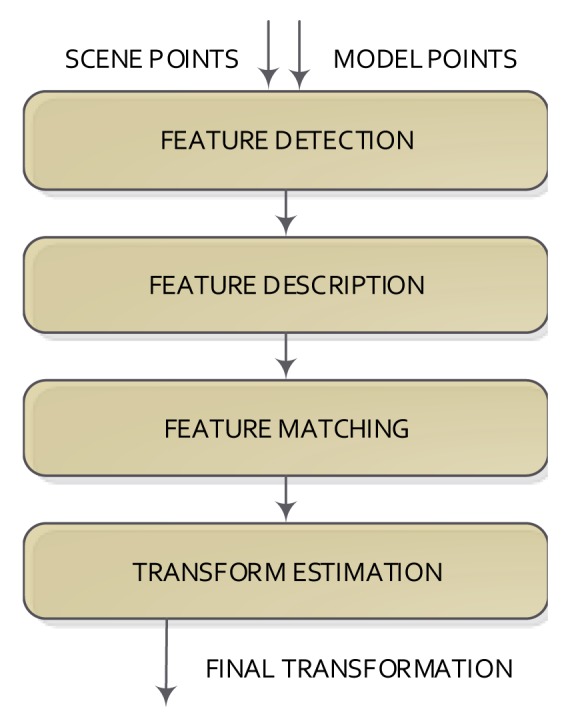
Basic scheme of the feature registration model.

In contrast to the methods based on the calculation of distances between pairs of points, the coarse methods commonly do not use all available data, but the downsampling of the data. Feature-based methods can be used, which try to reduce the amount of points from both sets (model and scene) using a given detection and description feature method to represent the input data. A feature has a position and a descriptor (the information around the position is described). Features can come from the image (visual features) or directly from 3D data (3D features). Irrespective of the kind of feature used, the steps of the registration methods are: feature detection/description, feature matching and transformation model estimation. These steps are shown in Figure 3.

The feature detection step tries to detect salient and distinctive parts of an object (shapes, closed regions, contours, lines, line intersections, *etc*.) in the datasets using a feature detection method and then represent the detected part as a set of values, normally called the feature descriptor. This step can be directly applied to the 3D data or to the 2D image (when using RGB-D cameras) and then assigning their 3D information. Thus, the number of elements is reduced extremely (from 340000 to less than 1000 in the case of a Kinect camera).

There are several feature detectors and descriptors that work with 2D data. One of the most used is the SIFT (scale invariant features transforms) [4], which provides both feature detection and description. The SURF feature (sped up robust features) [22] is similar, but faster than SIFT. For a deep study of the different visual features, see [23].

Only a few general purpose pure 3D feature detectors/descriptors have been presented. Some extensions of the well-known 2D Harris detector are proposed in [24]. A pure 3D descriptor is presented in [25]. It is called the fast point feature histograms (FPFH), and it is based on a histogram of the differences of angles between the normals of the neighbor points of the source point. Johnson [26] proposes a representation of a 3D surface for matching.

A planar patches feature extraction process is applied to the raw 3D data in order to obtain a complexity reduction in [27,28]. Koser and Koch [29] normalized RGBD data to a frontal view to obtain perspectively invariant surface features, while Wu *et al.* [30] rectified large planar regions to obtain perspective invariance, and Zeisl *et al.* [31] uses orthographic projection of RGB-D data to simplify matching.

The most used method for finding the transformation between correspondences is based on the RANSAC algorithm [10], since the matching step usually yields a lot of outliers. It is an iterative method that estimates the parameters of a mathematical model from a set of observed data, which contains outliers. In our case, we look for a 3D transformation (our model) which best explains the data (matches between 3D features). At each iteration of the algorithm, a subset of data elements (matches) is randomly selected. These elements are considered as inliers. A model (3D transformation) is fitted to those elements. Remaining data are then tested against the fitted model and included as inliers if its error is below a given threshold. If the estimated model is reasonably good (its error is low enough and it has enough matches), it is considered as a good solution. This process is repeated a number of times, and then, the best solution is returned.

Other registration methods are based on Genetic Algorithms (GA), as in [32]. Using these strategies, the problem of registration is dealt with as a search/optimization problem. The final transformation is generated using a genetic algorithm, getting some information of pairs to estimate the best transformation of all the transformations generated by the GA.

Stückler and Behnke [33] use a multi-resolution surfel (surface element) representation of the RGB-D images. This approach uses shape descriptors of the surfels similar to [25] and color histograms in a CIELAB (chromatic model close to the human perception) space. It uses these feature descriptors and the spatial properties of the multi-resolution octree to get the surfels correspondences. The method iterates to get the estimated transformation in a similar way to ICP.

### 2.2. Fine Methods

Fine registration methods are commonly used to refine a nearly close registration. In contrast to coarse methods, fine methods usually use all the available information in order to get the correct transformation between datasets. They usually follow an iterative method to incrementally refine the estimated registration due to the amount of data used. Nowadays, one of the most used methods is the iterative closest points [12,13] and its variants. Warping image methods were used in the past to register [34] colored stereo images. Color is used to constrain the search for the closest points in [35]. Recently, Kerl *et al.* [36] presented a 3D colored variation of the method in order to register consecutive RGB-D images.

#### 2.2.1. Iterative Closest Point and Variations

**Figure 4.**
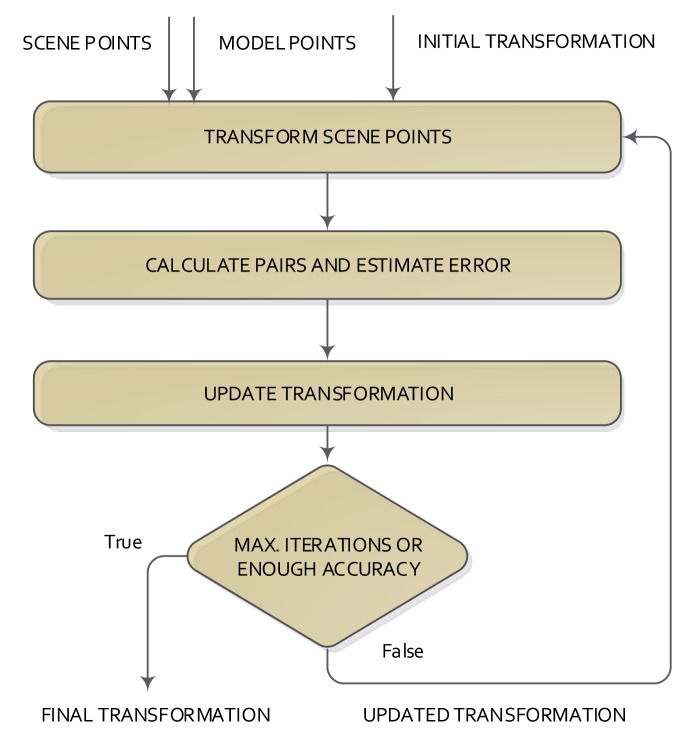
Basic scheme of the iterative closest point algorithm.

Some of the 2D/3D registration methods use the distance information between the matched points to calculate the global transformation that best explains the change of the position of two datasets. In this kind of method, the registration solves two problems iteratively: (1) finding the correspondence (or matching) between points; and (2) estimating the transformation that best explains the correspondences. The most used of these methods is the iterative closest point, which was introduced in [12,13]. The structure of the ICP method is shown in Figure 4.

One of the datasets is the model, and the other one is called the scene. The ICP starts with a given initial transformation and, then, continues iterating in two consecutive steps. First, scene points are processed using the current transformation. After that, the correspondence pairs are calculated using the scene and model points. At the end of each iteration and using the correspondence pair information, the transformation that best explained the correspondences is calculated. The base scheme of the ICP normally gets a local optimum solution for the registration, depending on the initial transformation given to the ICP method. This is the basic structure of the ICP, from which a lot of variations have emerged, which seek to change or improve any of the steps of the classical ICP.

The initial ICP [12] uses all of the points of the scene and model sets, matching the points with the least Euclidean distance. There are several methods that try to improve this time consuming step. For example, Turk [37] uses a uniform sub-sampling method to reduce the amount of points of the datasets. Another approach is the random selection of points [38], which quickly reduces the number of points, at the risk of losing some parts of the structure of the datasets. The method proposed by Weik [39] uses additional information to find the correspondences, like point color or intensity changes. Other methods use a kd-tree or a closest-point cache system [40] in order to speed up the search process. Other papers, like [41], weights the correspondence pairs with respect to their distance, giving less weight to pairs with higher distance or also depending on the difference between the normal of the points. Another criteria for weighting the matching step is to assign different weights with respect to the noise model of the sensors. For example, if it is known that the camera produces more noisy data for distant points, give them less weight than the closest points to the camera. Another approach [42] proposes rejecting a percentage of the worst matches, according to some criterion, usually distance from the points. Other variants, such as [38], reject the pairs whose distance is higher than a multiple of the standard deviation of the distances. Some papers, like [8,43], presented a collection of variations of the classical ICP in order to make it more robust and efficient.

Iterative closest methods are also used in current systems. The method proposed by Zhang *et al.* [44] searches multiple nearest points, then discards the correspondences with a larger distance than a computed threshold and only takes the correspondences that are bi-unique (i.e., only uses a correspondence if its model point has only one correspondence with the scene points).

In [17], Henry *et al.* present a hybrid approach of ICP and visual features. This modification of the original ICP makes use of SIFT visual features [4] and RANSAC [10] to get an initial guess transformation and then applies the ICP iterations, but instead of just getting the estimation that reduces the nearest neighbor distance of the points; it also uses the distances between the selected visual features using a parameter to weight both distances. This weighted system allows the ICP to align the two datasets using both the color of the visual features and the geometric information of the point cloud.

Other variations of the ICP are based on KinectFusion [19], which builds a model of the scene, while it computes the positions of the camera. This model is internally represented as a volumetric truncated signed distance function (TSDF). TSDF and the depth data integration comes from [45]. Each point in the space has stored the distance against the closest surface (positive if it is outside or negative if it is inside) and some weight value. This representation model allows the system to fuse the following depth images into one model, getting a smooth model. This registration against a model instead of the last frame images or point clouds allows the system to avoid the drift in the registration and gets smoother maps and camera trajectories compared to previous approaches.

Some different modifications of the original KinectFusion appeared. Kintinuous [46] presents a modification that allows the KinectFusion to work on bigger environments by shifting the model volume and saving the triangulation mesh that is removed from the working volume. In [47], they presented an integration of the Kintinuous with a color fast feature system based on [48]. Another extension of the Kintinuous [49] uses SURF features and constructs a pose graph to perform the loop closure to correct camera trajectories.

#### 2.2.2. Warping Image Methods

Warping image methods are based on the photo-consistency of the image pixels. Lucas-Kanade [34] presented a image registration method that uses the spatial intensity gradient at each point to modify the current estimated transformation and uses a Newton-Raphson iteration method to converge to better transformations. Following the same idea, Koch [50] used this approach to efficiently estimate the transformation of a textured model onto an image. This method is based on minimizing the photometric error between the observed and the synthesized image, where this synthesized image is generated by a 3D transformation of the 3D projected pixels of the image and then projecting again the transformed 3D points to a 2D image. Comport *et al.* [51] used this approach to estimate the camera position over consecutive stereo images due to the slightly movement of the camera. This approach has been adapted to RGB-D sensors in [20,52], where they show good registration results and camera position estimations using Kinect images. Recently, Kerl *et al.* [36] presented a refined approach of their dense visual odometry (DVO) method, where they use a weight system of residuals images (differences of the image's gradients) in order to gain robustness against noise and large residual values.

## 3. Comparison Framework

In this section, we present the comparison framework, including the considered scenarios (Section 3.1), to evaluate some of the aforementioned methodologies for different situations, and the metrics (Section 3.2) used to evaluate the results.

### 3.1. Considered Scenarios

In order to evaluate the different methodologies presented in the previous sections, we studied different scenarios that represent mainly the wide range of registration problems that can be considered for many applications, including full scene and small objects reconstruction. Moreover, we present here the specific methods we are going to evaluate that are the most relevant in this area.

#### 3.1.1. Scene Reconstruction

In order to register large scenes, several RGB-D datasets have to be registered into a common coordinate system. Most of the scene mapping methods use a simultaneous location and mapping (SLAM) [53–56] scheme in order to get the registration of consecutive RGB-D datasets. SLAM methods use a global rectification method in order to reduce the incremental error of the consecutive estimations.

We implemented some of the state-of-the-art registration methods in order to test and compare their results on representative scene RGB-D datasets. The methods tested are visual features, dense visual odometry and the KinectFusion. The visual features method is a hybrid system, which uses the Features from Accelerated Segment Test (FAST) detector [57,58] and the Binary Robust Independent Elementary Features (BRIEF) descriptor [59] and then a RANSAC algorithm to estimate the correspondences and the transformation between the features of scene and model datasets. In order to refine the final estimation results, we also applied an Iterative Closest Point algorithm. The FAST detector and the BRIEF descriptor are implemented in the OpenCV library (http://opencv.org/). The RANSAC and ICP methods are implemented in the Point Cloud Library (PCL) (http://pointclouds.org). We also implemented some variations of this method in order to see the difference of applying individually visual features or ICP to estimate the transformation. The dense visual odometry method is provided as a package in the Robot Operating System (ROS) system (http://vision.in.tum.de/data/software/dvo). Finally, the KinectFusion method is also provided by the PCL. KinectFusion was not originally implemented to register large scenarios, but PCL has a modification of this algorithm to extract the model as a polygon mesh and update the model.

In order to test the implemented scene mapping systems on large scenarios, we used the Technische Universität München (TUM) RGB-D dataset [60]. This dataset provides RGB-D and ground-truth data with the goal of evaluating the visual odometry and visual SLAM systems. The dataset contains the color and depth images of a Microsoft Kinect sensor along the ground-truth trajectory of the sensor. It provides images at a full frame rate (30 Hz) and sensor resolution (640 × 480). The ground-truth trajectory was obtained from a high-accuracy motion-capture system with eight high-speed tracking cameras (100 Hz). Further, it provides the accelerometer data from the Kinect.

**Figure 5.**
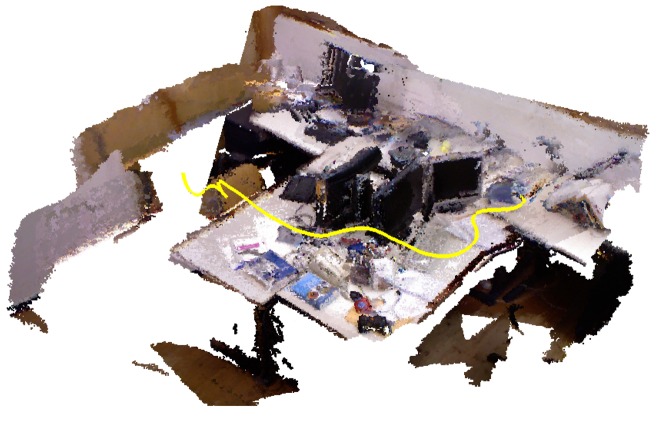
Example of the ground-truth point cloud reconstruction of the first 200 frames of the “fr1 desk” sequence; the yellow line represents the movement of the camera.

This original dataset contains 39 sequences recorded in two different scenarios. The “fr1” datasets are recorded on a typical office environment and the “fr2” datasets are recorded in a large industrial hall. Furthermore, some sequences are recorded using a hand-held Kinect and the rest using a Kinect mounted on a wheeled robot. Later, this dataset was extended with more sequences in order to test scenarios with different texture and structure appearances or scenes with dynamic objects. Figure 5 shows an example of the first 200 ground-truth positions of the camera as a yellow line and the reconstructed map.

Table 1 shows the average translation and rotation velocities of the different datasets. We observe that some datasets, like “fr1 xyz”, “fr2 xyz”, “fr2 desk” or all of “fr3”, have slow velocities. Other datasets, like “fr1 desk” and “fr1 desk2”, have high translations, so the movement between frames is higher. The “fr1 360” has slow translational velocity, but it has high rotational movement that also has influence on the registration results.

The authors concluded from their calibration measures that the relative error of the Kinect camera position on a frame-to-frame basis in the ground-truth data is lower than 1 mm.

#### 3.1.2. Small Object Reconstruction

The problem of object reconstruction is a well-known topic in computer vision [61], but with the appearance of the low-cost RGB-D sensors, a wide variety of approaches have been proposed. Object reconstruction covers from objects with big volumes, such as chairs or tables, to small and intricate ones, like plants, tools, *etc*. Related to the size of the object, the resolution of the RGB-D devices is an important aspect that affects the acquisition and, hence, the registration and reconstruction. The depth resolution expresses the minimum difference in depth that the camera is able to distinguish. The resolution is affected by the noise of the data. As the level of noise is increased, the performance of the registration methods decreases.

Regarding big elements, traditional algorithms could be used for registering the views, because each view has a large number of object points. Moreover, this sort of object is fairly described with visual and 3D features, making easier the pre-alignment by using RANSAC techniques for a coarse registration. Nevertheless, small objects have different aspects to be considered, i.e., size and geometry. Related to the size, object reconstruction is performed with a subset of the points. Irrespective of the size of the object, RGB-D sensors only work properly in a certain range of depth. Then, at least a minimum distance has to be preserved, and the object only will be represented with a part of the possible data. Thereby, the smaller the objects are, the less data that is available.

Another important issue is the object surface geometry. Due to the technique used by the low-cost RGB-D sensors, smooth surfaces are better estimated than rough ones. In scene mapping, the signal-to-noise ratio (SNR) is high due to the fact that scenes usually have big smooth surfaces (roof, floor, walls, etc.) that are well extracted, and then, the registration methods obtain a good transformation to align the views. However, objects do not always follow this kind of geometry. Normally, big objects have regions of smooth surfaces, except those intricate ones, such as trees. Nevertheless, when the target is small, less smooth surfaces appear; therefore, the SNR decreases. Hence, traditional techniques cannot be applied directly to the point cloud.

In this section, five different registration techniques are tested for object reconstruction acquired using RGB-D sensors:

- Coarse registration (Section 2.1): a feature-based approach has been evaluated using RANSAC. The number of features in objects is small compared to the scene case. 3D features are time-consuming techniques, and also, due to the noise of the RGB-D sensors, they are not usually reliable; hence, visual features are more often used. We use the SIFT feature extraction and description, as it is one of the most used algorithms in object reconstruction. RANSAC is used to estimate the best translation that registers the SIFT descriptors of two views.
- Fine registration (Section 2.2): in this section, the registration is performed with the well-known iterative closest point. In particular, the Chen and Medioni ICP variant with edge rejection has been tested. In order to be able to use fine registration directly, several views, close to each other, are registered.
- A combination of coarse and fine registration methods is applied to evaluate a common process of pre-alignment and refinement.
- A well-known implementation for reconstruction with RGB-D sensors is KinectFusion [19,62]. This method was developed for environments, not for objects. It tends to smooth the objects by using a truncate signed distance function (TSDF) and a model of the scene, which makes shapes rounded or even disappear when they are too small.
- The last presented method is the RGBDemo [21], which has been specifically developed for object reconstruction using RGB-D sensors. It uses color markers (ARToolKit markers) to make an initial coarse registration. Once the initial alignment is done, an ICP and then a subsampling process return the final result.

**Figure 6.**
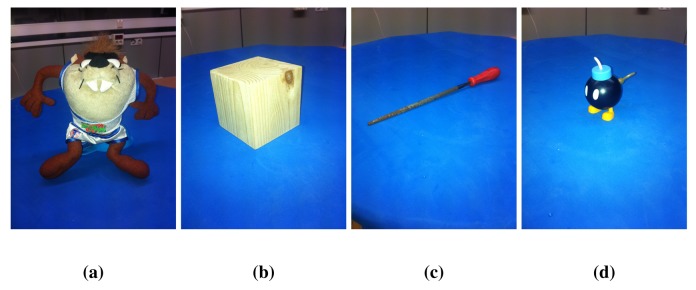
Objects used for experimentation. (**a**) Taz toy; (**b**) cube; (**c**) tool; and (**d**) bomb toy.

Figure 6 shows different objects with specific features associated with their shapes, which affect the registration. The dataset has been created for this experimentation, due to the lack of a dataset of objects acquired using RGB-D sensors, where KinectFusion and RGBDemo results are presented. They have been acquired using a Microsoft Kinect on a turntable. Three hundred twenty views have been acquired of each element around them (about 1.13 degrees per step) in order to use the fine registration method without pre-alignment. The distance that separates sensor and objects is a meter, and the camera is placed diagonal-upper to allow the markers of RGBDemo to be visible. The first one, Figure 6a, is a Taz toy of 15 cm in height with a large variety of colors. The second object (Figure 6b) is a wooden cube of 8 *cm*^3^, which has faces where a knot appears and others, less varied in color. In the third column, a tool (Figure 6c) is presented. It is 30 *cm*-long; the thinnest part is 0.5 *cm*, and the widest is 2.8 *cm*. The last object is a bomb toy shown in Figure 6d with 8 *cm* height and 5 *cm* width. It has different colors, thin parts, such as the white one on the top, the body part with a smooth curve and the back part with a key attached.

A previous segmentation of the region of interest has been performed in order to isolate the object. A segmentation combining color and depth information has been used to extract the object from the scene for ICP and RANSAC algorithms. The background of the scene has been carefully established using blue chroma. Moreover, the distance from camera to object is previously known. Regarding RGBDemo, the method uses a white floor with specific markers (printed in white paper) to localize the space where the object is placed. RGBDemo uses only that region in the registration process (markers for coarse registration and ICP for fine registration). However, KinectFusion works with the whole data supplied by the camera having as a consequence two different motions: moving parts as the object on the turntable and static parts as the rest of the scene.

### 3.2. Metrics and Performance Measures

As previously mentioned, we used the TUM RGB-D dataset [60] for scene reconstruction. This dataset proposes some evaluation measures based on the comparison of the estimated trajectories of the camera and the ground-truth ones.

The relative pose error measures the local accuracy of the trajectory over a fixed time interval, Δ. Therefore, the relative pose error corresponds to the drift of the trajectory, which is useful for the evaluation of visual odometry systems. The dataset authors define the relative pose error at time step *i* as:

(3) $$E_{i}:={\left(Q_{i}^{-1}Q_{i+\Delta }\right)}^{-1}(P_{i}^{-1}P_{i+\Delta })$$

From a sequence of *n* camera poses, we obtain in this way *m* = *n* − Δ individual relative pose errors along the sequence. From these errors, they propose to compute the root mean squared error (RMSE) over all time indices of the translational component as:

(4) $$\text{RMSE}(E_{1:n},\Delta ):={\left(\frac{1}{m}\sum_{i=1}^{m}||\text{trans}(E_{i})|{|}^{2}\right)}^{1/2}$$

where *trans*(*E_i_*) refers to the translational components of the relative pose error, *E_i_*. The time parameter, Δ, needs to be chosen. For visual odometry systems that match consecutive frames, Δ = 1 is an intuitive choice; *RMSE*(*E*_1:_*_n_*) then gives the drift per frame that we will use to measure the quality of the implemented systems.

For object reconstruction, we will use visual appearance analysis, due the non-existence of a common dataset and its correspondent ground-truth.

## 4. Results and Discussion

In this section, we present the results of the experiments done for both scene and object reconstruction. For each part, we discuss the obtained results.

### 4.1. Scene Reconstruction

We have performed two different experiments to evaluate scene registration methods. In the first one, we analyze the results of each method on the different scene sequences. In order to improve clarity, some “y-axes” are trimmed, because there are some high error values that represent a totally misalignment or registration error. Moreover, some graphs are not complete, since the implementation fails, and it is not able to recover or register the following frames. The blue lines represents the translational error. The ground-truth translational magnitude (in meters) is included on the following graphics as red lines and reflects the relative error with respect to the real translation. Following the considerations of the dataset authors’ evaluation method, we do not show the rotational error, because the camera is in continuous motion; an error in the rotation estimation involves an error in the translational error.

**Figure 7.**
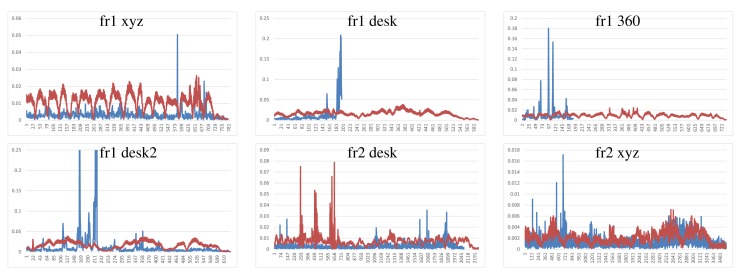
Blue: KinectFusion relative pose translational errors (y-axis, meters) on the selected datasets (x-axis, frames). Red: relative ground-truth motion (meters).

Figure 7 shows the results of the KinectFusion implementation. We observe that KinectFusion fails in the “fr1 desk” and “fr1 360” datasets. The rest of the results looks quite smooth, despite some high errors. Some of these datasets have parts where there is a lack of geometry, so the method gets a high error or it gets lost. According to the real relative movement of the camera represented with red lines, the “fr1 xyz” dataset has continuous changes in velocity and direction. In the “fr2-desk” dataset, the camera described sudden/abrupt movements. These movements are caused by a lack of ground-truth information, so the distance between “consecutive” frames is relatively high.

**Figure 8.**
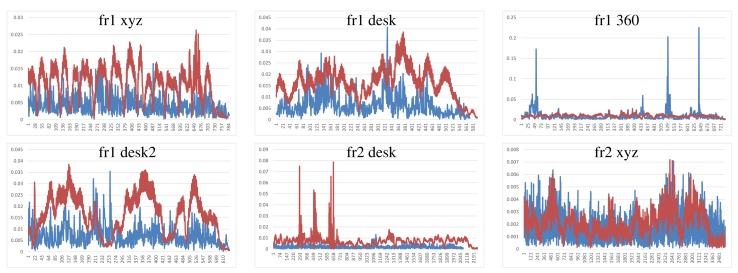
Dense visual odometry relative pose translational errors (y-axis, meters) on the selected datasets (x-axis, frames).

Dense Visual Odometry results are shown in Figure 8. We observe that DVO has more variability in the “fr1 xyz” caused by sudden direction changes. DVO gives good results on the datasets with smooth movements, like “fr2 desk”. The “fr1 360” dataset presents high errors caused by the high rotational camera motion. In general, the DVO method works better than the KinectFusion, except on the “fr2 xyz”.

**Figure 9.**
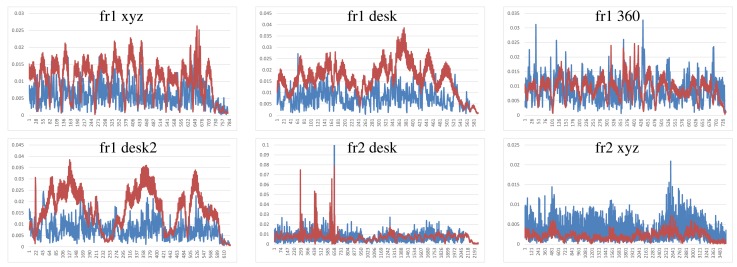
Visual features + ICP relative pose translational errors (y-axis, meters) on the selected datasets (x-axis, frames).

Visual Features with ICP refinement results, showed in Figure 9, have mostly the same or higher errors as the ones in DVO and KinectFusion. There are no relatively high errors on most of the datasets. The “fr2 desk” dataset shows higher error in one of the last frames (Frame 659). The “Fr2 xyz” dataset presents a high error due to the error introduced by the localization of visual features, which is not corrected by the ICP refinement.

**Figure 10.**
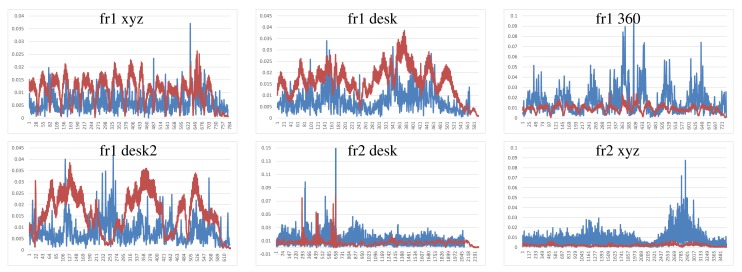
Visual features relative pose translational errors (y-axis, meters) on the selected datasets (x-axis, frames).

The results of the visual features method (Figure 10) show a similar structure than the visual + ICP method, but the errors are higher in almost all the cases. We now observe the difference of applying the ICP refinement step. In general, the visual features method without the ICP refinement has higher errors.

**Figure 11.**
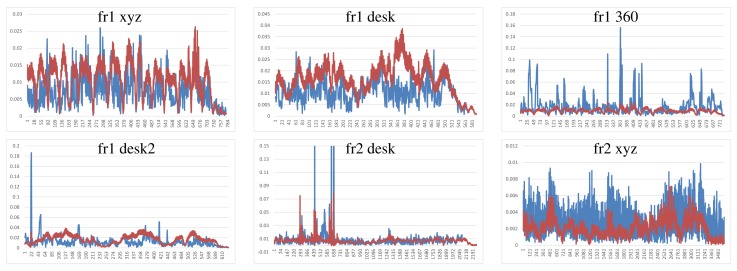
ICP relative pose translational errors (y-axis, meters) on the selected datasets over all the frames (x-axis, frames).

Figure 11 shows the results of the iterative closest point method. We observe similar errors as the ones obtained with the visual features method, but it also has some high errors, like at the beginning of the “fr1 desk2” and in the “fr2 desk”. As expected, ICP gets better results than visual features in the datasets with less movement, since the frames to register are initially very close. In the “fr1 360” dataset with high rotational movements, the ICP gets several high errors. In general, we can observe that the visual features with ICP refinement works better than both methods individually. The implementations of the visual features and ICP methods have no correction, and that is why high errors are obtained that make no sense for a particular application, such as scene reconstruction. These errors can be detected and corrected or discarded by a simply movement boundary limitation, like the one that KinectFusion and DVO use.

**Figure 12.**
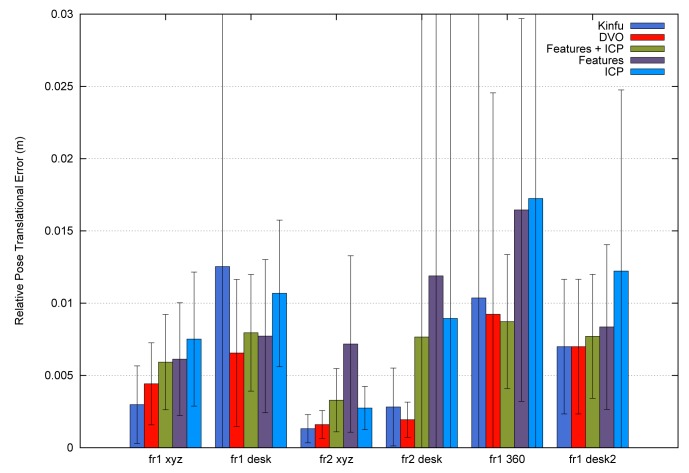
Error means (bars) and the standard deviations (error lines) of the five different methods applied to the selected datasets.

To summarize, Figure 12 shows the average errors and the standard deviation of the five tested systems on the different datasets. We observe that the datasets with lower velocities (“fr1 xyz” and “fr2 xyz”) have less error values. In “fr1 360”, we spot the influence of the rotational velocities on the registration methods. Despite it having a low translational velocity (0.21 m/s), its rotational velocity increases the error values of the methods, as can be observed in the error bars and their standard deviation. In general, we observe the improvement of the visual features method with the ICP refinement. This improvement is particularly noticeable in the “fr1 360” dataset. We conclude that the dense visual odometry method is one of the most robust methods, and it provides low errors.

**Figure 13.**
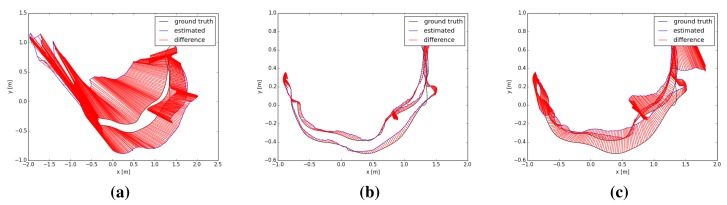
Trajectories estimated on the dataset “fr1 desk2” by the KinectFusion (**a**), the dense visual odometry (**b**) and the visual features + ICP (**c**) methods.

Finally, Figure 13 shows the error in the camera pose of the KinectFusion, DVO and visual features + ICP methods over the “fr1 desk2” dataset. Due to the incremental estimation of the camera pose, a high error in one estimation can lead to a totally misaligned trajectory. This is the case of the KinectFusion. When a high error is detected, an additional method should be implemented in order to discard the frame and use the previous correct position.

In the second experiment, we focus on the analysis of the results of the three main tested methods in scenarios with special features. The TUM dataset has a special “fr3” set of scenes where different combinations of texture and geometry appearance are presented. We use four different combinations of texture and structure appearance. The first column of Table 2 shows some of the used scenes. Table 2a represents a scene with detailed texture and geometry appearance. Table 2b shows an empty and white floor, so it represents a non-texture and non-structure information dataset. Table 2c only has some posters in a wall, so it represents the texture and non-structure information situation. The last dataset, Table 2d, represents the non-texture and structure information situation.

To improve the interpretation of the results we did not include the ICP and visual feature methods in this experiment. Furthermore, the first experiment showed that the combined features with ICP refinement worked better than the two methods separately.

Table 2 shows the relative pose translational errors of the three tested methods. The results show a general poor registration, since most of the errors are higher than the real translational movement. However, the errors are mostly lower than 2 cm. Despite the simplicity of the scenes, we observe a high error in most of the scenes with different methods. In the datasets with a lack of structure information Table 2b,c, the results of the three methods are similar. In general, we observe that the KinectFusion method gets the best results with the exception of the dataset with texture, but no structure information, where the visual features with ICP refinement gets the best results.

**Figure 14.**
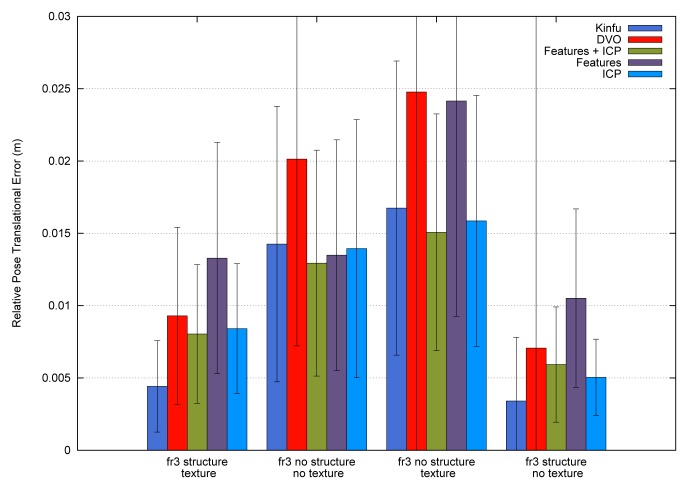
Error means (bars) and the standard deviations (error lines) of the five different methods applied to the different texture and structures datasets.

Figure 14 shows the average errors and the standard deviation of the five tested methods on the different datasets. We can observe that the dense visual odometry method is getting the biggest errors on these datasets. The KinectFusion method (Kinfu) gets the best results on the datasets with geometry information and gets results closest to the best on the other two datasets.

### 4.2. Small Object Reconstruction

**Figure 15.**
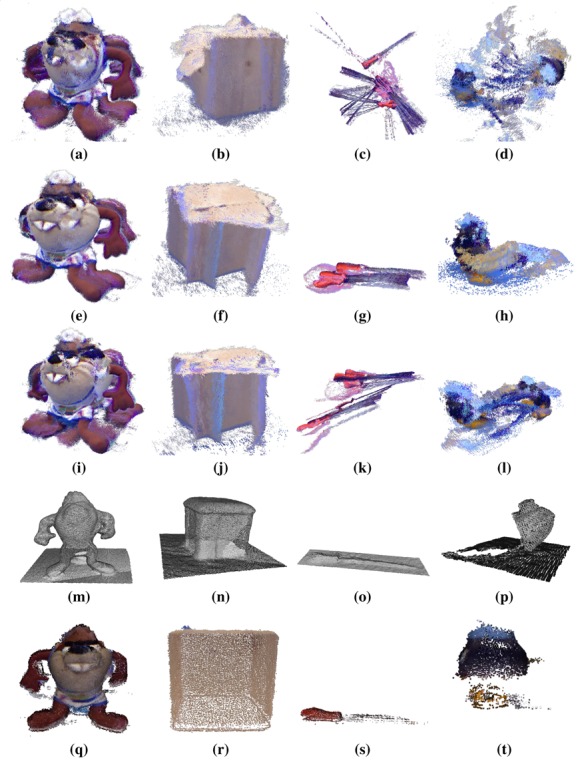
Object registration results of the tested methods. First row: RANSAC with SIFT features registration. Second row: ICP registration. The third row shows the result of the RANSAC and ICP combination. The fourth row presents the results of KinectFusion. Finally, the fifth row presents the RGBDemo results.

For small object reconstruction, we have made an experiment using some representative methods of objects in Figure 6. Figure 15 shows the result of the different registration algorithms. The first row has the coarse registration results; the second one presents the ICP results. In the third, a combination of coarse pre-alignment and fine registration using the RANSAC and ICP methods is shown. The fourth row presents KinectFusion's results. Lastly, Figure 15q–t shows the RGBDemo results.

Figure 15a–d shows the coarse registration of the objects with RANSAC and visual SIFT features. Figure 15a has been well registered in the front part, where a large variety of colors appear. However, the error in the back part produces the wrong final result. The cube in Figure 15b shows some views that are bent, caused by features in the top matched with others in the lateral part, due to the similarity, causing a bad registration. In Figure 15c,d, the error in the registration is caused because these shapes have few features, and it is not possible to register them properly.

The fine registration ICP applied to the test objects (Figure 15e–h) shows that, in general, the results are better than in coarse registration, but still with a considerable error. These errors are caused by the low SNR, mainly in thin or sharp parts, where the RGB-D sensor cannot return the depth information accurately.

The registration results of combining (Figure 15i–l) pre-alignment using RANSAC and refinement with ICP shows how if the wrong pre-alignment is achieved, ICP cannot return a proper registration.

**Figure 16.**
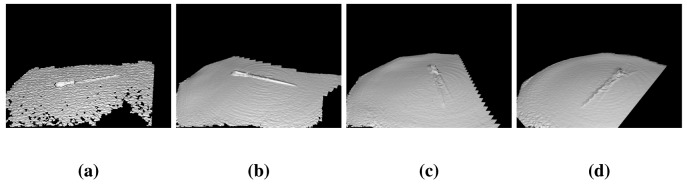
Different moments of the tool reconstruction with KinectFusion. The objects is mixed progressively with the floor.

KinectFusion results show that it does not work properly in object reconstruction. For example, the cube in Figure 15n, where the edges of the shape are rounded. In addition, thin parts close to each other are joined, making them distorted. This method uses only 3D information; hence, in objects, such as the bomb toy, where the shape is rounded, the method cannot put the different views in the right place. Finally, the tool (Figure 15o) is joined to the floor, due to its thin geometry. Figure 16 shows different moments of the reconstruction, where step by step, the algorithm mixes the object and the floor.

**Figure 17.**
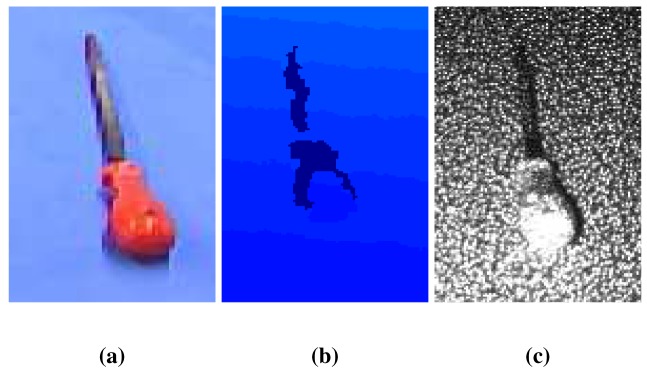
The color, depth and infra-red images obtained by the camera. Using the infra-red speckles, the depth is obtained with a structured light technique. In this case, the object has thin parts that not enough speckles reach, and no depth information can be obtained.

RGBDemo algorithm results are presented in Figure 15q–t. The Taz toy (Figure 15q) and cube (Figure 15r) are well registered. The cube has the edges rounded due to the subsampling. The tool in Figure 15s is well registered in the thickest part, but the thin part disappeared, due to different aspects. One is the subsampling, but the main reason is the way in which low-cost RGB-D sensors recover the depth information. They use a correlation window (http://wiki.ros.org/kinect calibration/technical) to estimate the depth information. In case the window of 9 × 7 speckles (they use speckle pattern) had more points in the floor than in the object, the floor depth information will be dominant. Figure 17 shows the color (Figure 17a), depth (Figure 17b) and infra-red (Figure 17c) images of the tool acquired with the RGB-D sensor. The depth image has no information, because the speckles that reach the object are not enough to estimate the profundity. The bomb toy in Figure 15t is properly registered at the top part, but the low part of the body is incomplete. This is produced by self-occlusion. RGBDemo uses visual markers, which have to be always visible. In case the full object has to be fully reconstructed, it should be acquired from different positions, and finally, all registration results aligned together.

According to the four objects that have been selected to evaluate different aspects of the registration methods, the analysis shows that as the Taz toy is the largest object and has several colors, enough visual features can be found for the RANSAC algorithm. It also has a varied geometry what allows ICP-based methods to align the views properly. Despite these features, the traditional methods (RANSAC, RANSAC + ICP and ICP) cannot achieve an accurate final result, due to the cumulative error between views, producing the final wrong closure. KinectFusion obtains a reasonable result, despite the fact that it joins several parts, due to the smoothing characteristics of the method.

The cube is a low textured object, making difficult the feature extraction in some of its faces. Then, both methods based on RANSAC cannot achieve good results. On the other hand, despite the geometry being simple, the RGB-D sensors provide noisy data, mainly in edges, which makes it difficult to evaluate the point cloud correspondence and general transformation between two consecutive views. For that reason, the ICP cannot obtain a final proper registration. KinectFusion tends to smooth surfaces, because it uses a model of the scene and a TSDF method for point-to-model correspondence estimation, which produces the rounded edges.

The tool has been selected due to the thin and low textured shape. Neither enough features could have been extracted nor are there geometry aspects suitable for traditional algorithms. Thus, RANSAC and ICP cannot register the views. KinectFusion does not work properly due to the aforementioned smoothing characteristics of the method, producing a non-accurate final reconstruction.

Finally, the bomb toy is a small object with different areas. There are parts having color feature areas and others with varied geometry. These features produce good results for RANSAC when the featured parts are registered, but fail in the rest. On the other hand, ICP works properly with the back part, where a keypoint is attached to the object, but fails when the round part of the Bomb is aligned. KinectFusion cannot achieve a good result due to the lack of geometry.

Different conclusions can be extracted analyzing the above experimentation results. In general, regarding the size of the object, all methods find more difficulties in registering views from small objects, because either few features appear or the noise becomes more relevant. That is the reason why the best result in general terms is achieved by the RGBDemo algorithm, using external visual markers in order to help the registration. The coarse alignment is based on these visual markers; then the lack of texture does not affect it, and the fine registration works properly, because of the good coarse pre-alignment. For thin and small objects, such as the tool and the bomb toy, a slight filtering process should be used in order to avoid over-rejection of data when those objects are commonly originally represented with few data. Despite the RGBDemo presenting good results, there is still work to do in order to allow different angles of the sensor against the object to minimize self-occlusions. There exist new proposals focused on this problem, such us [63], where a model-based multi-view registration method is presented for 3D markers to allow the registration of objects.

## 5. Conclusions

In this paper, we have first made a description of different registration methods using low-cost RGB-D sensors, dividing them into coarse and fine methods. This classification facilitates the description of the main features of the algorithms. Then, we have developed an experimental validation of different registration methods in two representative applications: scene and object reconstruction.

For scene registration, we have tested five different registration methods and quantitatively measured the error in the pose estimation using a state-of-the-art RGB-D dataset for visual odometry and SLAM systems. Using the evaluation measures and tools provided by the dataset, we analyzed the results of the tested methods, which are the KinectFusion, the dense visual odometry, the ICP, a visual feature-based method and visual features with the ICP refinement method. Results showed that the DVO method gets the lowest registration error, and it is the most robust. KinectFusion does not work properly with datasets where frames have a lack of geometry.

For object registration, we have tested also five different registration methods and qualitatively measured the error using a new RGB-D dataset created for this evaluation; specifically, a visual feature-based method using SIFT and RANSAC, the ICP variant of Chen and Medioni, the visual features with ICP refinement method and the new methods, KinectFusion and RGBDemo. KinectFusion is one of the most used methods for object reconstruction. However, RGBDemo is a very different approach, due to the use of ARToolKit markers. Results show that traditional algorithms did not provide accurate results, while visual marker-based methods obtain better registrations. Different areas of research still remain a challenge in this topic, such as better techniques focused on registering objects acquired with low-cost RGB-D sensors.

As future work, we plan to continue comparing new methods and analyzing new RGB-D sensors for these problems. Moreover, quantitative techniques to evaluate object registration error have to be enhanced, since they are the most used for object registration and reconstruction visual inspection.

## Figures and Tables

**Table 1.** The number of frames and the average translation and rotation velocities of the used datasets.

**Dataset**	**Number of Frames**	**Average Translational Velocity (m/s)**	**Average Rotational Velocity (deg/s)**
fr1 xyz	780	0.24	8.92
fr1 desk	595	0.41	23.33
fr1 360	739	0.21	41.6
fr1 desk2	631	0.43	29.31
fr2 xyz	3,594	0.06	1.72
fr2 desk	2,234	0.19	6.34
fr3 structure and texture	904	0.193	4.323
fr3 no structure and no texture	451	0.196	2.712
fr3 no structure and texture	447	0.299	2.890
fr3 structure and no texture	791	0.166	4.00

**Table 2.** Relative pose translational errors in blue color (y-axis, meters) on the fr3 datasets (x-axis, frames) of the dense visual odometry, KinectFusion and visual features with ICP refinement methods. Red color values represent the ground-truth pose movement. Images belong to the dataset; (**a**) structure and texture. (**b**) no structure and no texture. (**c**) no structure and texture. (**d**) structure and no texture.

**Example Image**	**Dense Visual Odometry**	**KinectFusion**	**Visual Features** + **ICP**
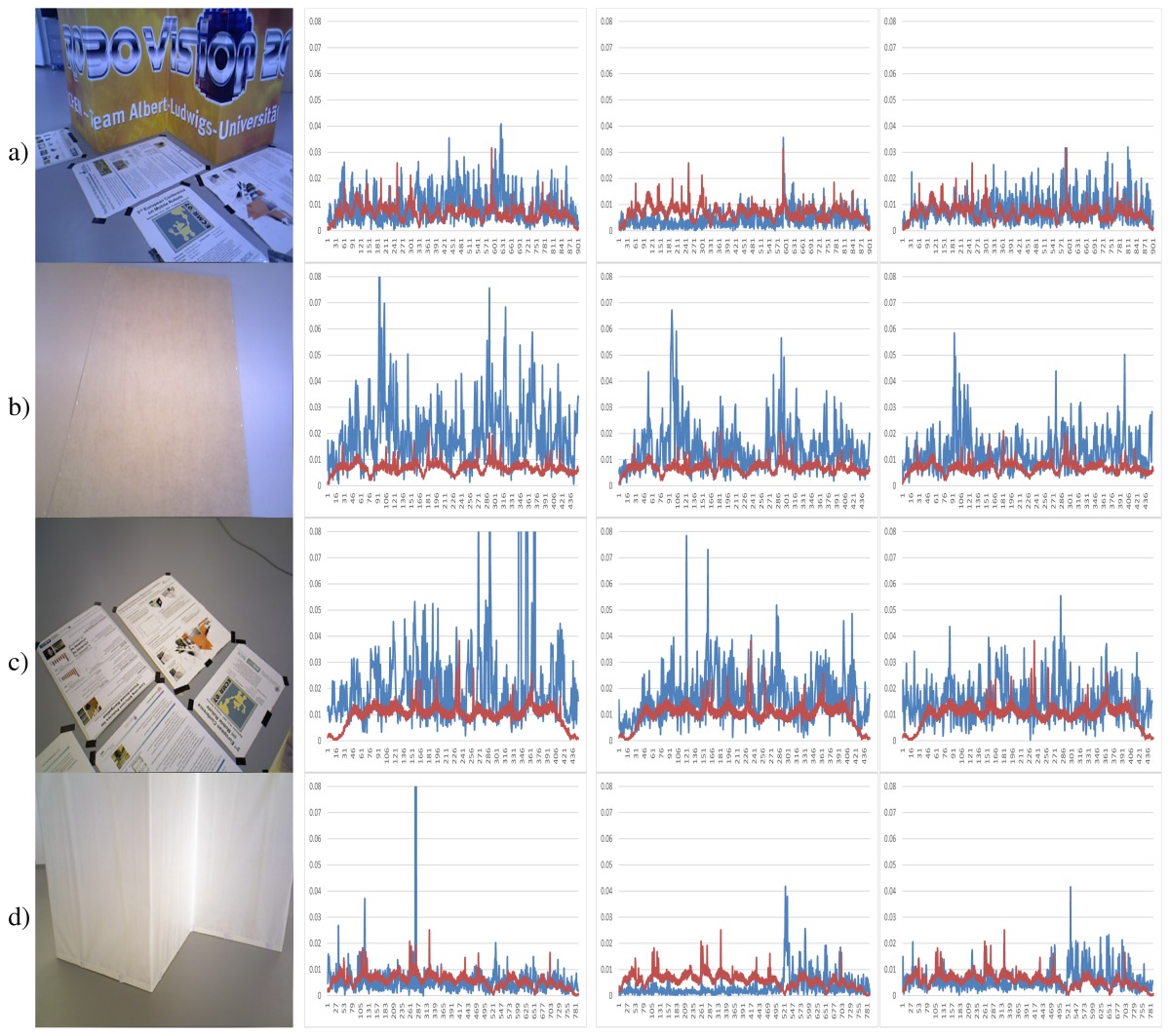			

## References

[1] Yang F., Ding M., Zhang X., Wu Y., Hu J. (2013). Two Phase Non-Rigid Multi-Modal Image Registration Using Weber Local Descriptor-Based Similarity Metrics and Normalized Mutual Information. Sensors.

[2] Duan L., Guan T., Yang B. (2009). Registration Combining Wide and Narrow Baseline Feature Tracking Techniques for Markerless AR Systems. Sensors.

[3] Cazorla M., Viejo D., Pomares C. Study of the SR4000 camera.

[4] Lowe D.G. (2004). Distinctive Image Features from Scale-Invariant Keypoints. Int. J. Comput. Vis..

[5] Khoshelham K., Elberink E.O. (2012). Accuracy and resolution of kinect depth data for indoor mapping applications. Sensors.

[6] Zitov B., Flusser J. (2003). Image registration methods: A survey. Image Vis. Comput..

[7] Tam G., Cheng Z.Q., Lai Y.K., Langbein F., Liu Y., Marshall D., Martin R., Sun X.F., Rosin P. (2013). Registration of 3D Point Clouds and Meshes: A Survey from Rigid to Nonrigid. IEEE Trans. Vis. Comput. Gr..

[8] Rusinkiewicz S., Levoy M. Efficient variants of the ICP algorithm.

[9] Pomerleau F., Colas F., Siegwart R., Magnenat S. (2013). Comparing ICP variants on real-world data sets. Auton. Robots.

[10] Fischler M.A., Bolles R.C. (1981). Random sample consensus: A paradigm for model fitting with applications to image analysis and automated cartography. Commun. ACM.

[11] Hetzel G., Leibe B., Levi P., Schiele B. 3D object recognition from range images using local feature histograms.

[12] Chen Y., Medioni G. Object modeling by registration of multiple range images.

[13] Besl P., McKay N. (1992). A method for registration of 3-D shapes. IEEE Trans. Pattern Anal. Mach. Intell..

[14] Salvi J., Matabosch C., Fofi D., Forest J. (2007). A review of recent range image registration methods with accuracy evaluation. Image Vis. Comput..

[15] Campbell R.J., Flynn P.J. (2001). A Survey of Free-form Object Representation and Recognition Techniques. Comput. Vis. Image Underst..

[16] Crum W.R., Hartkens T., Hill D.L.G. (2004). Non-rigid image registration: Theory and practice. Br. J. Radiol..

[17] Henry P., Krainin M., Herbst E., Ren X., Fox D. (2014). RGB-D mapping: Using depth cameras for dense 3D modeling of indoor environments. Experimental Robotics.

[18] Raguram R., Frahm J.M., Pollefeys M. A Comparative Analysis of RANSAC Techniques Leading to Adaptive Real-Time Random Sample Consensus.

[19] Izadi S., Kim D., Hilliges O., Molyneaux D., Newcombe R.A., Kohli P., Shotton J., Hodges S., Freeman D., Davison A.J. (2011). KinectFusion: Real-time 3D reconstruction and interaction using a moving depth camera.

[20] Steinbrucker F., Sturm J., Cremers D. Real-time visual odometry from dense RGB-D images.

[21] Kramer J., Burrus N., Echtler F., Parker M., Herrera C.D. (2012). Object Modeling and Detection. Hacking the Kinect.

[22] Bay H., Ess A., Tuytelaars T., Gool L.V. (2008). Speeded-up Robust Features (SURF). Comput. Vis. Image Underst..

[23] Gil A., Mozos O., Ballesta M., Reinoso O. (2010). A comparative evaluation of interest point detectors and local descriptors for visual SLAM. Mach. Vis. Appl..

[24] Gomb P., Kurzynski M., Wozniak M. (2009). Detection of Interest Points on 3D Data: Extending the Harris Operator. Computer Recognition Systems 3.

[25] Rusu R., Blodow N., Beetz M. Fast Point Feature Histograms (FPFH) for 3D registration.

[26] Johnson A. (1997). Spin-Images: A Representation for 3-D Surface Matching. PhD Thesis.

[27] Viejo D., Cazorla M. 3D Model Based Map Building.

[28] Viejo D., Cazorla M. (2014). A robust and fast method for 6DoF motion estimation from generalized 3D data. Auton. Robots.

[29] Koser K., Koch R. Perspectively Invariant Normal Features.

[30] Wu C., Clipp B., Li X., Frahm J.M., Pollefeys M. 3D model matching with Viewpoint-Invariant Patches (VIP).

[31] Zeisl B., Köser K., Pollefeys M. Automatic Registration of RGBD Scans via Salient Directions.

[32] Brunnstrom K., Eklundh J., Uhlin T. (1996). Active Fixation for Scene Exploration. Int. J. Comput. Vis..

[33] Stückler J., Behnke S. Model Learning and Real-Time Tracking Using Multi-Resolution Surfel Maps.

[34] Lucas B.D., Kanade T. An Iterative Image Registration Technique with an Application to Stereo Vision.

[35] Druon S., Aldon M.J., Crosnier A. Color Constrained ICP for Registration of Large Unstructured 3D Color Data Sets.

[36] Kerl C., Sturm J., Cremers D. Robust Odometry Estimation for RGB-D Cameras.

[37] Turk G., Levoy M. Zippered polygon meshes from range images.

[38] Masuda T., Sakaue K., Yokoya N. Registration and Integration of Multiple Range Images for 3-D Model Construction.

[39] Weik S. Registration of 3-D partial surface models using luminance and depth information.

[40] Simon D.A. (1996). Fast and Accurate Shape-Based Registration. PhD Thesis.

[41] Godin G., Rioux M., Baribeau R. Three-dimensional registration using range and intensity information.

[42] Pulli K. Multiview registration for large data sets.

[43] Zinsser T., Schmidt J., Niemann H. A refined ICP algorithm for robust 3-D correspondence estimation.

[44] Zhang L., Choi S.I., Park S.Y. Robust ICP Registration Using Biunique Correspondence.

[45] Curless B., Levoy M. A Volumetric Method for Building Complex Models from Range Images.

[46] Whelan T., Kaess M., Fallon M., Johannsson H., Leonard J., McDonald J. Kintinuous: Spatially Extended KinectFusion.

[47] Whelan T., Johannsson H., Kaess M., Leonard J., McDonald J. Robust Real-Time Visual Odometry for Dense RGB-D Mapping.

[48] Huang A.S., Bachrach A., Henry P., Krainin M., Maturana D., Fox D., Roy N. Visual Odometry and Mapping for Autonomous Flight Using an RGB-D Camera.

[49] Whelan T., Kaess M., Leonard J., McDonald J. Deformation-based Loop Closure for Large Scale Dense RGB-D SLAM.

[50] Koch R. (1993). Dynamic 3D Scene Analysis through Synthesis Feedback Control. IEEE Trans. Pattern Anal. Mach. Intell..

[51] Comport A., Malis E., Rives P. Accurate Quadrifocal Tracking for Robust 3D Visual Odometry.

[52] Audras C., Comport A., Meilland M., Rives P. Real-time dense RGB-D localisation and mapping.

[53] Dissanayake M., Newman P., Clark S., Durrant-Whyte H., Csorba M. (2001). A solution to the simultaneous localization and map building (SLAM) problem. IEEE Trans. Robot. Autom..

[54] Endres F., Hess J., Engelhard N., Sturm J., Cremers D., Burgard W. Evaluation of the RGB-D SLAM System.

[55] Chang P., Shen J., Cheung S.C. A Robust RGB-D SLAM System for 3D Environment with Planar Surfacess.

[56] Shen J., Su P.C., Cheung S., Zhao J. (2013). Virtual Mirror Rendering With Stationary RGB-D Cameras and Stored 3-D Background. IEEE Trans. Image Process..

[57] Rosten E., Drummond T. Fusing points and lines for high performance tracking.

[58] Rosten E., Drummond T. Machine learning for high-speed corner detection.

[59] Calonder M., Lepetit V., Ozuysal M., Trzcinski T., Strecha C., Fua P. (2012). BRIEF: Computing a Local Binary Descriptor very Fast. IEEE Trans. Pattern Anal. Mach. Intell..

[60] Sturm J., Engelhard N., Endres F., Burgard W., Cremers D. A Benchmark for the Evaluation of RGB-D SLAM Systems.

[61] Chane C.S., Schtze R., Boochs F., Marzani F.S. (2013). Registration of 3D and Multispectral Data for the Study of Cultural Heritage Surfaces. Sensors.

[62] Newcombe R.A., Izadi S., Hilliges O., Molyneaux D., Kim D., Davison A.J., Kohli P., Shotton J., Hodges S., Fitzgibbon A.W. KinectFusion: Real-time dense surface mapping and tracking.

[63] Saval-Calvo M., Azorin-Lopez J., Fuster-Guillo A. Model-Based Multi-view Registration for RGB-D Sensors.

